# Dr. Putnam

**Published:** 1843-06

**Authors:** 


*Obituary.*—Dr. Putnam, surgeon dentist of New Orleans, but formerly of Nashville Tennessee, where, for many years he exercised the duties of his profession, died the 31st of March inst. at Vicksburg Mississippi.

Also, more recently, in the city of Natchez, Miss. Mr. Irwin Taylor, student of dental surgery, younger brother to Drs. James, Edward and Joseph Taylor. He was a young gentleman of high aspirations and attainments, and gave promise, had his life been spared, of eminent usefulness in the profession for which he was preparing.

